# Transmaterial Becoming

**DOI:** 10.1080/13528165.2016.1223453

**Published:** 2016-10-20

**Authors:** Klaus Spiess, Lucie Strecker

*The Hour of the Analyst Dog* is a collaborative performance project by the Medical University and the University of Applied Arts in Vienna that closed *The Play on the Burden of Representation*, a contemporary art exhibition commemorating the seventy-fifth anniversary of Freud’s death held at the Belvedere/21er Haus in Vienna.

**Figure:**
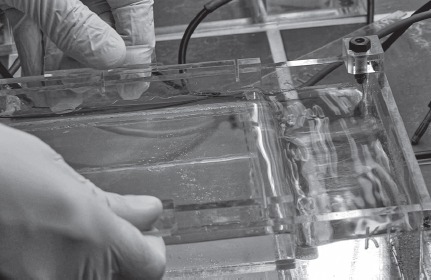
Department of Cell Biology/Genetics, University Salzburg 2015

*© Spiess/Strecker*

Our work melded resources conserving biological and cultural memory: a museum archive, a genetic lab and a biobank. We connected these resources’ practices – all relying on recalling the past in the present. Relevant to cloning, psychoanalysis and reenactment is their reanimation of historical DNA, transferring affects or gestures from the past to a context of the present.

We began our undertaking by harvesting hairs from Sigmund Freud’s dog Jofie, woven into a rug by his daughter Anna. Using these hairs we sequenced one of the dog’s zeitgeber genes, and cloned it to become a synthetic timer in blood cells from a living psychoanalyst. The body heat emitted by the audience at the exhibition caused the resynchronization of – and consequently produced fluorescence in – the cells, signalling the end of the performance.

Transgenetics became an arena for performative enquiry: How could a relic that brings a past/history back to life gain agency without turning an open future into the inevitable repetition of copies? How could this transgenetic being act without evoking archaic fears of a reversal in chronological order (Schneider 2001: 96)?

What theories are relevant when we enable an audience to bring a dog-human hybrid back to life?

We critically applied the findings of linguist Judith Roof – that geneticists falsely ascribe DNA an arbitrary relationship to matter (Roof 2007: 54–5) – in conjunction with Jacques Derrida’s concept of grafting as the power of reopening closed texts (Kakoliris 2015).

Would such a transgenetic alliance enable us to reopen Freud’s retroactive constructivism and linear determinism of the past and future towards a more open-ended concept of drive, dreams and the unconscious?

Here we adopted the concept of transmaterial becoming to account for the way time constitutes itself by actions and doings between matter and technologies as well as human and non-human performers/actants.

**Figure:**
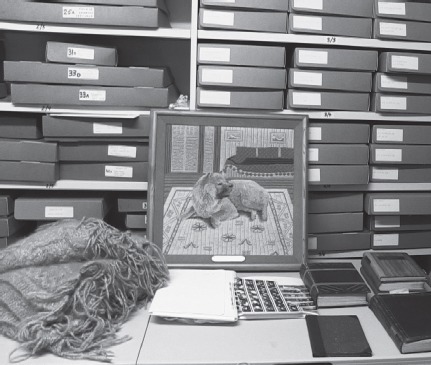
Sigmund Freud Museum, London 2014

*© Spiess/Strecker*

**Figure:**
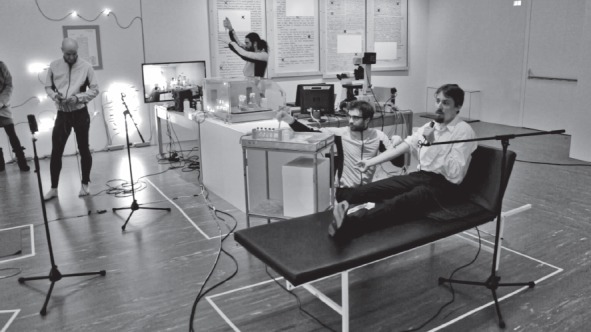
21er Haus/Belvedere, Vienna 2015

*© Spiess/Strecker*

To initiate a transmaterial mnemonic process we enabled organic and bioinorganic matter to ‘live differently in time’ (Landecker qtd in Bruyère 2015: 133) using a biotechnological process consisting of heating, freezing, cloning and immortalizing bioinorganic and biological matter. In a second step, we transplanted an inanimate outside into the animate inside of a living cellular organism to introduce a discrepancy between the past, present and future – in our view, a prerequisite of the transmaterial mnemonic becoming (Barker 2015: 53).

Prior to the lab work we explored the Freud family’s relevant commemorative and archiving practices: descriptions of the dog’s behaviour documented by Freud’s analysands, Anna’s birthday letters to Freud in Jofi’s name, and embroidery depicting the dog – all ascribing specific figurative commemorative features to Jofi.

When we visited Freud’s Marshfield Gardens house in London, to our delight we were led to a small attic where an inconspicuous handwoven rug was stored in a grey archive box next to Freud’s deathbed. The wool was interwoven with Jofi’s water-repellent dog hair, a once common application. While documenting on video the conservation of sufficient hairs, we recalled Foucault’s reading of the archive: its tendency towards revivification – the zombification of its artefacts – which is precisely what constitutes its inherent vitality.

A key element of our concept is the dog’s regular state of high awareness at Freud’s sessions, synchronously reacting to the patients’ unconscious affects. This capacity of the dog’s resulted in a mutual exchange between Freud and his pet that determined when the individual sessions terminated (Grinker 1997).

To nourish this informed dog hair’s inherent capacity, we sequenced Jofi’s Timeless Gene (hTIM, Locus Chr 12q12-q13, Chromosome 10; NC_006592.3*)* to transplant it into white blood cells from our cooperating psychoanalyst. We immortalized these cells to create a molecular timer that would cue the end of the exhibition: the spectators’ combined body heat during a meditation session was transmitted by handheld sensors to resynchronize the psychoanalyst’s cells in a bioreactor on display in the space. These cells had been running chaotically, out of sync due to the transplanted genes from Jofi. The final outcome was fluorescence in the resynchronized cells, heralding the closure of the commemorative exhibition.

**Figure:**
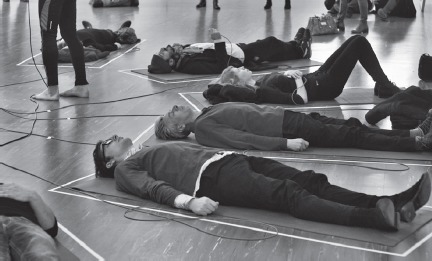
21er Haus/Belvedere, Vienna 2015

*© Spiess/Strecker*

Beyond its genealogical, legal, collective or taxonomical definition, we substituted the figure of the dog with the molecular timer, which subsequently became the canvas on which its transmaterial, commemorative portrait emerged (Bruyère 2015: 130).

We understood drive in its distinct, cloned manifestation as a ‘subjectless subjectivity of technology’, as an avatar-like agency proceeding without an agent (Apter 2008: 8.2.5.) – not in Lacanian terms, as a ‘subjectivization without a subject’ (Bains qtd in Apter 2008: 8.2.7.), where the dog would be seen as the drive itself.

By merging conservative categorizations of history-prone, male and human, and of presentprone, female and dog, and the spectators’ body heat – the latter understood as an ‘associated milieu’ and an ontological tool – we imbue transgenetics with biopolitical and queer potential: Deleuzian multiplicity.

This ‘subjectless subjectivity of technology’ closed the exhibition as an independent transformer. It transpired both beyond the uncanny copies produced by cloning, beyond anthropocentric predisposition. This transformer acted as a trickster by imposing distance on conventional norms of psychoanalytical and biological time, adding to pre-existing psychoanalytical sensitivities.

Genetically inevitable DNA reading faults and random DNA errors are to be expected in transspecies cloning. As such, going beyond simple copying, this material glitch transgresses the DNA’s past in favour of an open-ended, potent future for the transplanted organisms – a strong feature behind evolution.

The emergence of transmaterial becoming suggests that the inanimate bioinorganic matter – constitutive for both living matter and for chemical processes – of the nucleic acids of Jofi’s DNA could not develop their temporal becoming without a living host, nor could the accelerated molecules of the spectators’ heat flow develop on their own. As such, without being united with the psychoanalyst’s living cells, both would remain a mass of highly determined actions and reactions. At the same time, the blood cells of the host would live in a continuous present with no discernible past or future, had a bioinorganic input from the outside world not challenged this similarity of instants and thereby engendered multiplicity. It is this transmaterial mnemonic encounter that occurs in what Deleuze calls life’s immanent flow of becoming: the ‘splitting of time’ (Barker 2015: 57).
